# Physiological and Neurobehavioral Disturbances Induced by Al_2_O_3_ Nanoparticle Intoxication in Nile Tilapia Fish: Benefits of Dietary Chamomile Essential Oil

**DOI:** 10.1155/2023/6700708

**Published:** 2023-06-03

**Authors:** Mayada R. Farag, Chuntian Zheng, Heba S. A. Gharib, Enas EL-Hady, Eman A. A. Mahdy, Azza M. A. Abo-Elmaaty, Shimaa M. Abou-Zeid, Mahmoud Alagawany, Alessandro Di Cerbo, Mahmoud M. Azzam, Abdulaziz A. Al-Abdullatif, Mona A. Hassan

**Affiliations:** ^1^Forensic Medicine and Toxicology Department, Veterinary Medicine Faculty, Zagazig University, Zagazig 44519, Egypt; ^2^Institute of Animal Science, Guangdong Academy of Agricultural Sciences, Key Laboratory of Animal Nutrition and Feed Science (South China) of Ministry of Agriculture, State Key Laboratory of Livestock and Poultry Breeding, Guangdong Public Laboratory of Animal Breeding and Nutrition, Guangdong Key Laboratory of Animal Breeding and Nutrition, 510640 Guangzhou, China; ^3^Behaviour and Management of Animal, Poultry and Aquatics Department, Faculty of Veterinary Medicine, Zagazig University, 44511, Egypt; ^4^Anatomy and Embryology Department Veterinary Medicine Faculty, Zagazig University, Zagazig 44519, Egypt; ^5^Pharmacology Department, Faculty of Veterinary Medicine, Zagazig University, Zagazig 44519, Egypt; ^6^Department of Forensic Medicine and Toxicology, Faculty of Veterinary Medicine, University of Sadat City, Sadat City 6012201, Egypt; ^7^Poultry Department, Faculty of Agriculture, Zagazig University, Zagazig 44519, Egypt; ^8^School of Biosciences and Veterinary Medicine, University of Camerino, Matelica, Italy; ^9^Department of Animal Production College of Food & Agriculture Sciences, King Saud University, Riyadh 11451, Saudi Arabia

## Abstract

Despite the usage of nanoparticles (NPs) is rapidly increasing, several experts have noted the risk of their release into ecosystems and their potential negative impacts on biological systems. However, the available studies on the neurobehavioral impacts of aluminum oxide nanoparticles (Al_2_O_3_NPs) on aquatic organisms are little. Hence, this study targeted to ascertain the harmful effects of Al_2_O_3_NPs on behavioral characteristics and genotoxic and oxidative damages in Nile tilapia fish. In addition, the beneficial role of chamomile essential oil (CEO) supplementation in reducing these effects was also investigated. In the current study, fish were distributed into 4 equal groups (*n* = 60 fish per group). The control group was fed a plain diet only, the CEO group received a basic diet complemented with CEO at a level of 2 mg/kg diet, the ALNP group received a basic diet and was exposed to an approximate concentration of 1/10th LC_50_ of ALNPs nearly 5.08 mg/L, and the combination group (ALNPs/CEO group) received a basal diet coadministered with ALNPs and CEO at the aforementioned percentages. The findings revealed that *O. niloticus* exhibit neurobehavioral changes along with changes in the level of GABA, monoamines in the brain tissue, and serum amino acid neurotransmitters, besides a reduction of AChE and Na^+^/K^+^-ATPase activities. In addition to brain tissue oxidative damage with upregulation of proinflammatory and stress genes, such as HSP70 and caspase-3, supplementation of CEO significantly reduced the negative impacts of ALNPs. These results showed that CEO has neuroprotective, antioxidant, genoprotective, anti-inflammatory, and antiapoptotic properties in fish that have been exposed to ALNPs. Therefore, we advise its usage as a valuable addition to fish diet.

## 1. Introduction

Recently, applications of nanotechnology have increased in aquaculture ranging from vaccine delivery and nutrients to health management, pollution remediation, water purification, and breeding of fish [1]. Aluminum, zinc, iron, and silicon nanomaterials or their oxide forms make up a significant portion of the worldwide market for engineering products. The global market for aluminum oxide nanoparticles (Al_2_O_3_NPs) stated that Al_2_O_3_ is a widespread type of nanoparticles (NPs) used for different industrial purposes. Aluminum-based nanoparticles represent 20% of all nanosized materials used in a very broad range of applications [2]. Due to their widespread usage and unintentional environmental releases, nanoparticles' negative impacts on the aquatic environment are receiving particular attention despite their beneficial qualities.

The concentration of aluminum oxide nanoparticles (Al_2_O_3_NPs) in the marine environs is at nanogram levels, but the possible hazard of Al_2_O_3_NP in the aquatic ecosystem has been demonstrated in earlier investigations. Nogueira et al. [3] stated that Al_2_O_3_NPs have hazardous effect on *Daphnia magna* in sublethal levels. Moreover, because of their high surface reactivity, NPs have both acute and chronic detrimental impacts on aquatic species, including conformational changes, chromosomal mutation, and oxidative damage [4]. Conversely, there is little scientific evidence about the behavior and neurotoxicological aspects of Al_2_O_3_NP, and the overall impacts of NPs on the aquatic environment remain unclear.

Fish are the most widely used screening species because they are included in marine toxicology investigations. They are in the food chain directly consumed by humans and serve as indicators of the quality of the water and the marine environment [5]. Because of a variety of characteristics, including its high tolerance to diseases and stress, prolific rate, ease of reproduction in incarceration, and lastly good environmental tolerance to different circumstances, *Oreochromis niloticus* is a significant species in aquaculture around the world and a reliable biological model that is used frequently in toxicological research [6]. Numerous researches have displayed that nanoparticles can target the central nervous system since nanoparticles may significantly harm different types of brain cells [7, 8]. Nanoparticle toxicity results in the formation of reactive oxygen species at the cellular level, which can harm macromolecules including DNA, proteins, and fat metabolism and cause oxidative damage [9–12]. Chamomile (*Matricaria chamomilla L*.) is a useful medicinal plant in Europe and Asia and belongs to the *Asteraceae* family. Since chamomile is mostly produced in Argentina, Egypt, Hungary, and Poland, it is one of the most popular and commonly used herbal remedies. It is a highly fascinating industrial plant, and its raw materials are broadly applied in agricultural, cosmetic, and pharmaceutical applications due to the extensive number of biologically active ingredients found in the blooms [13]. *Matricaria chamomilla* have a variety of biological properties, including neuroprotective efficacy against oxidative stress caused by cerebral ischemia/reperfusion damage in rats. In vitro tests revealed that chamomile had strong antiplatelet and anticarcinoma activity in addition to mild antibacterial properties [14]. Chamomile is used to treat gastrointestinal discomfort, coughing, common cold, fever, wounds, and skin disorders including psoriasis, eczema, and bronchitis. Moreover, it is abundant in flavonoids, which are powerful antioxidants to combat free radicals [15]. The pharmacological properties of chamomile essential oil include antibacterial, carminative, sedative, antiproliferative, anti-inflammatory, and antioxidant effects [16] Nile tilapia is the most common and predominant cultured fish; in this work, the median lethal concentration (96 hours LC_50_) of AL_2_O_3_NPs in Nile tilapia fish was evaluated. It also sought to investigate the effects of aqueous exposure to AL_2_O_3_NPs at sublethal concentrations in *O. niloticus* fish, including neurotoxic impacts, and to evaluate the role of dietary supplementation with CEO in reducing the toxic insult of AL_2_O_3_NPs via investigating the neurologic and behavioral parameters in addition to stress and inflammatory cytokine-related gene expression.

## 2. Material and Methods

### 2.1. Aluminum Nanoparticle Preparation and Characterization

**Figure 1:**
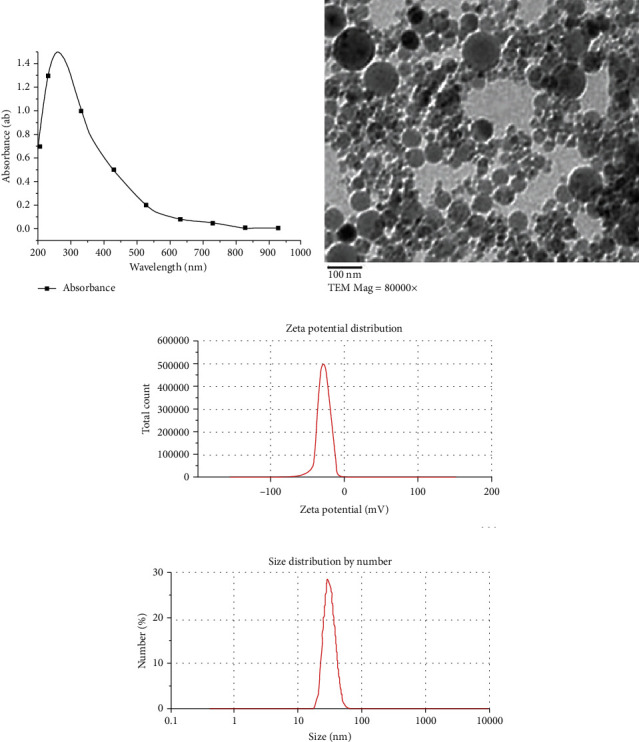
The characterization of ALNPs. The results showed the maximum peak at 330 nm (a). AgNPs under TEM were spherical with 55-80 nm average size (b). The net surface charge was -25 mV (zeta potential analysis) (c). The exact size was 70 nm (DLS analysis) (d).

*Pseudomonas fluorescens* MT46 isolate was inoculated in nutrient broth medium followed by incubation at 37°C for 24 h. Then, the supernatant was centrifuged for 15 min at 6000 rpm. 25 mL of bacterial supernatant was added to 75 mL of AL_2_O_3_ (0.5 mM), and then, the optimum conditions were determined as pH 7, temperature 35°C, concentration of 0.5 mM, 24 h reaction time, and 200 rpm agitation speed. The obtained aluminum nanoparticles were characterized with four advanced devices: UV-vis spectrophotometer, transmission electron microscopy (TEM), dynamic light scattering particle size, and zeta potential analyzer (studying the behavior of nanoparticles in suspension with regard to charge and size using a technique called dynamic light scattering (DLS)); the outcomes were as follows: the spherical aluminum nanoparticles, absorbed UV at 330 nm, TEM showed a spherical shape with an average size of 55 nm to 80 nm, a charge -25 mV, and a size 70 nm (Figure 1). The preparation and characterization of nanoparticles were conducted according to the standard method adopted by the Microbiology Department, Agriculture Faculty, Zagazig University, Egypt.

### 2.2. Tested Oil

Chamomile essential oil (CEO) was obtained from Natural Oils ElHawag Company, Cairo, Egypt. Gas chromatography mass spectrometry (GC-MS) investigation was used to ascertain the active ingredients found in the CEO used in the current investigation [17]. The GC-MS analysis detected a total number of 17 active ingredients in the CEO with different peak areas (%) and retention times as shown in Table 1.

### 2.3. Acute Toxicity Study

A total of 60 *O*. *niloticus* fish, weighing 50 ± 0.15 g, were divided into 6 groups of 10 fish each for detection of LC_50_ of ALNPs. They were taken from the Fish Hatchery of El-Abbassa, Al-Sharkia, Egypt, in good health. They sustained a 14-day acclimation time in glass aquarium filled with dechlorinated tap water. To reduce the absorption of NPs in food and the excretion of feces, fish were not given food throughout the testing period. To maintain a steady nanoparticle concentration, the NP-treated water was replaced every 24 hours. Without the addition of any stabilizing agents, various suspension concentrations of ALNPs were made and dispersed using a bath sonicate for 1 hour just before usage. Following range finding tests of Temiz and Kargın [5] and for the lethal toxicity investigation, a graded series of ALNP suspension in 0, 10, 20, 40, 80, and 160 mg/L concentrations was administered in triplicate. Then, in a 30 L aquarium with 20 L of the NP-treated water, ten fish were exposed to each concentration for 96 hours alongside a control fish (fish reared in distilled water free from NPs). For determination of the LC_50_, daily records of the fish mortality, swimming propensities, and exterior morphology for each group were made in comparison with the control group. Finney's probit approach was used to get the value of 96 h LC_50_ for ALNPs with 95% confidence intervals [18].

### 2.4. Experimental Fish and Formulation of the Tested Diets

240 acclimatized *O*. *niloticus* fish, each weighing 50 ± 0.12 g, were used. They consumed a specially designed basic diet, without the tested ALNPs or CEO supplement, at 5% rate of the fish biomass, three times each day. The American Public Health Association's [19] guidelines for recommended water quality were followed. Additionally, all aquariums were adjusted to the same rearing circumstances, which included temperature, pH, dissolved O_2_, and ammonia with a regulated photoperiod of 10 hours of light and 14 hours of darkness.

The CEO was added to the constituents of the basic diet as presented in Table 2 at a rate of 2 gm/kg diet (based on a preliminary study in our lab), and all of the diet's components were mechanically blended, pelletized, and dried thoroughly in the air for one day at 25°C before being kept at 4°C till being fed to fish.

Nile tilapia fish were distributed into 4 equal groups with 3 replicates per group (20 fish per replicate). Each replicate was housed in glass aquarium measuring 100 × 50 × 40 cm and holding dechlorinated tap water (160 L). The first group was housed in glass aquarium with clean water and fed a basic diet deprived of the tested supplement. The second group received a basic diet complemented with CEO at a rate of 2 mg/kg diet (CEO group). The third group received a basic diet and was exposed to 1/10th LC_50_ of ALNPs (5.08 mg/L). The fourth group (ALNPs/CEO group) received a basal diet coadministered with ALNPs and CEO at the aforementioned levels.

Throughout the 30-day experiment, fish were fed 3 times/day (at 7:00 a.m., 11:00 a.m., and 4:00 p.m.), and feed intake was changed every 2 weeks in accordance with fish weight increase.

### 2.5. Effect of Treatments on Behavioral Pattern

In the last week of the experiment, comfort and aggressive behaviors were observed in all treated groups in each aquarium (one observation per day) so as to evaluate the behavioral responses of *O. niloticus* after exposure to ALNPs and CEO supplementation and represent them in percentages based on the number of fish. All observations were made by a single observer and carried out in constant period from 7:00 a.m to 9:00 a.m.

There are several categories of comfort behavior, such as chafing, schooling, resting, surfacing, and eliminative behavior [20–22]. The agressive behaviour including fin tuging (biting), chasing and mouth pushing were also observed [20, 23].

#### 2.5.1. Sampling of Blood and Brain Tissue

At the trial end, fishes were sampled, cleansed with methanol, and anesthetized with 200 mg/L tricaine solution (MS-222). Blood was obtained from the caudal blood arteries by means of sterilized syringes and without the use of an anticoagulant. The serum was collected by blood centrifuging at 1075 × *g* for 20 min. The levels of amino acid neurotransmitters were determined using the obtained sera. The spinal cord section was used to scarify fish, and samples from the brain were subsequently removed and homogenized on ice in 50 volumes (*v*/*w*) of phosphate-buffered saline (PBS) at pH 7.2 followed by centrifugation for 15 min at 15,000 rpm at 4°C, and the supernatants were stored at -20°C to be used for biochemical analysis and evaluation of antioxidant markers. The concentration of total protein was estimated in tissue homogenate by protein assay kit (23225, Thermo Fisher Scientific, Waltham, MA, USA). Another brain sample was promptly putted in liquid nitrogen and then held at -80°C till the extraction of total RNA. Another group of specimens was preserved at -20°C till utilized. The experimental procedures were conducted as approved by the Institutional Animal Care and Use Committee of Zagazig University (ZU-IAURC). All biosafety measures were applied throughout the whole research and after finishing the experiment. All equipment and surfaces were disinfected. Fish and blood samples were decontaminated before discarding.

#### 2.5.2. Assays of Antioxidant and Oxidative Damages

The brain was homogenized to assess the antioxidant markers such as CAT (catalase), SOD (superoxide dismutase), and GSH (reduced glutathione) levels according to the colorimetric method established by Aebi [24], Nishikimi et al. [25], and Beutler [26], correspondingly. The methodology designed by Ohkawa et al. [27] was used to colorimetrically analyze malondialdehyde (MDA) as a lipid peroxidation marker. Using a colorimetric kit (no. 10005020; Cayman's Chemical Co., Ann Arbor, USA), the protein carbonyl (PC) content was assessed.

The extent of DNA oxidative damage in the experimental fish's brain tissues was assessed by the comet assay [28]. Fifty individual cells were investigated per slide. A CCD camera (Olympus, Japan) coupled to the fluorescence microscope (Zeiss Axiovert Inc., Germany) captured images of the cells under investigation. For each cell, tail DNA percentage and tail lengths (the lengths of the DNA migration tails) were calculated. Additionally, the tail moment scores from each cell's comet picture were estimated using the Comet Assay Project software.

#### 2.5.3. Neurochemical Analysis

*(1) Detection of Neurotransmitters in the Brain and Serum Amino Acids*. Reverse-phase HPLC was used to detect the concentrations of norepinephrine (NE), dopamine (DA), serotonin (5-HT), and *γ*-aminobutyric acid (GABA) in brain homogenate using C-18 column and electrochemical detector. The mobile phase was methanol/PBS (3 : 97, *v*/*v*) with a flow rate of 1 mL/min. The capacity of injections was 20 liter, and 270 nm was the detecting wavelength. By using the external standard procedure, the neurotransmitter concentrations were assessed using serial dilution injection from the standards and peak area detection. Plotting peak were compared areas against the relative levels to establish a linear standard curve [29, 30].

The neurotransmitter amino acids (glycine, glutamine, and aspartate) were estimated in serum by using the fluorescence detections of the analyte's o-phthalaldehyde/2-mercaptoethanol derivatives. Then, reversed-phase HPLC and precolumn derivatization were completed their separation [31].

*(2) Determination of AChE and Na^+^/K^+^-ATPase Enzyme Activities*. According to the steps mentioned by Ellman et al. [32], the spectrophotometric evaluation of AChE activity was performed at 412 nm. By means of a modified version of Svoboda and Mosinger's [33] approach, the activity of Na^+^/K^+^-ATPase was determined [34].

#### 2.5.4. Quantitative Real-Time PCR

The expression of inflammatory and stress genes was assessed as described in our previous study [12]. TRIzol reagent (easyREDTM, iNtRON Biotechnology, Korea) was used to extract the total RNA from the frozen brain samples. Then, extracted RNA was used to synthesize the first-strand cDNA by a QuantiTect® Reverse Transcription kit (Qiagen, Germany). The forward and reverse primers for targeted genes are as follows: SOD (F: 5-GACGTGACAACACAGGTTGC-3 and R: 5-TACAGCCACCGTAACAGCAG-3), CAT (F: 5-TCAGCACAGAAGACACAGACA-3 and R: 5-GACCATTCCTCCACTCCAGAT-3), GSH-Px (glutathione peroxidase) (F: 5-CCAAGAGAACTGCAAGAACGA-3 and R: 5-CAGGACACGTCATTCCTACAC-3), P53 (tumor suppressor protein) (F: 5-GCATGTGGCTGATGTTGTTC-3 and R: 5-GCAGGATGGTGGTCATCTCT-3), caspase-3 (F: 5-GGCTCTTCGTCTGCTTCTGT-3 and R: 5-GGGAAATCGAGGCGGTATCT-3), HSP70 (heat shock protein 70) (F: 5-CTCCACCCGAATCCCCAAAA-3 and R: 5-TCGATACCCAGGGACAGAGG-3), IL-1*β* (interleukin 1-beta) (F: 5-CAAGGATGACGACAAGCCAACC-3 and R: 5-AGCGGACAGACATGAGAGTGC-3), TNF-*α* (tumor necrosis factor-*α*) (F: 5-GGAAGCAGCTCCACTCTGATGA-3 and R: 5-CACAGCGTGTCTCCTTCGTTCA-3), IFN-*γ* (interferon gamma) (F: 5-AAGAATCGCAGCTCTGCACCAT-3 and R: 5-GTGTCGTATTGCTGTGGCTTCC-3), and *β*-actin (F: 5-CAGCAAGCAGGAGTACGATGAG-3 and R: 5-TGTGTGGTGTGTGGTTGTTTTG-3). The qPCR analysis was done in a Rotor-Gene Q instrument with a QuantiTect® SYBR® Green PCR kit (Qiagen, Germany) under the following thermocycler condition: 95°C for 10 min, followed by 40 cycles of 95°C for 15 s, 60°C for 30 s, and 72°C for 30 s. The comparative 2^-*ΔΔ*Ct^ method established by Livak and Schmittgen [35] was used to detect the relative mRNA expression patterns for each gene.

### 2.6. Determination of Aluminum Residues

1 gram of brain tissue was subjected to digestion in 4 mL of the digestion solution (1 nitric: 1 perchloric acid) for 24 h at room temperature, then heated at 110°C for 2 h then be cooled and deionized water was added. Then the digested tissues be warmed for 1 h in a water bath. Then be filtered, and 25 mL of deionized water was added. Then aluminum residues were determined by flame atomic absorption spectrophotometer (FAAS).

### 2.7. Statistical Analysis

One-way analysis of variance (ANOVA) was used to statistically evaluate all of the data using SPSS (version 20.0, SPSS Inc., USA). The means of the groups were compared using Tukey's multiple comparisons post hoc test, where the statistical significance was accepted at *p* < 0.05. Means ± SE (standard error) was used to express the examined data.

## 3. Results

### 3.1. LC_50_ Value of ALNPs and Behavioral Responses in Exposed Fish

**Figure 2:**
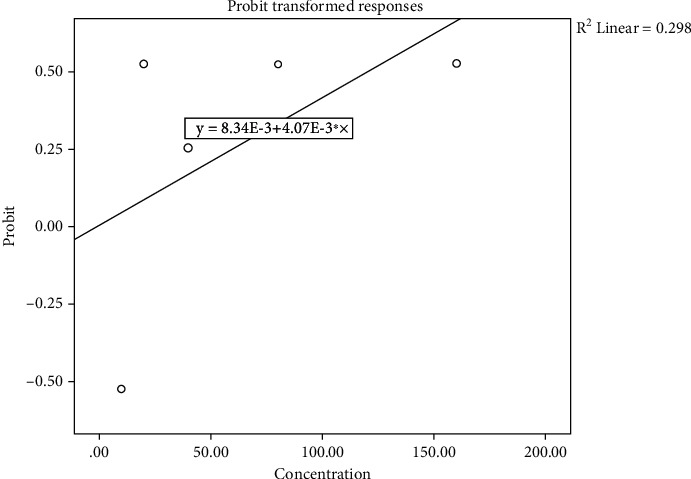
Probit analysis of 96 h LC_50_ calculation of ALNPs.

The 96 h LC_50_ value for ALNPs in fish was determined to be 50.82 mg/L as presented in Figure 2. When monitored over a 96-hour period, it was demonstrated that fish mortalities increased as ALNP levels increased. Fish exposed to the lowest concentration of ALNPs (10 mg/L) as well as control fish showed no clinical symptoms during the exposure period, whereas abnormal clinical signs were observed instantaneously in those exposed to higher concentrations (>10 mg/L). In the ALNP-intoxicated fish at 10 mg/L, respiratory distress and air-gulping were observed, whereas other diseased conditions such as sluggish movement, uncoordinated swimming, and hyperventilation were noticed at concentration of ≥20 *μ*g/L as depicted in Table 3.

### 3.2. Behavioral Impairments in *O. niloticus* Fish

Table 4 shows that fish exposed to ALNPs (1/10 LC_50_, 5.08 mg/L), for 30 days, exhibited substantial levels of discomfort as seen by declines in the observed chafing, resting, and schooling behaviors, whereas surfacing and elimination rates were constant across all treatment groups. Additionally, these fish had a high level of aggression, as seen by an increase in pursuing and mouth pushing behaviors, with no discernible alterations in fin-pulling behavior in comparison with the control. The administration of CEO to the ALNP groups modified the comfort behavior indices, decreased the amount of aggression relative to the ALNP group, and restored the overall level of aggression back to normal.

### 3.3. Antioxidants, Oxidative Stress, and DNA Damage Variables

Compared to the control, CAT, SOD, and GSH activities were dramatically reduced in the ALNP group, whereas GSH and CAT activities were greatly enhanced by the coadministration of CEO with ALNPs, and their values were returned to normal range.

PC and MDA levels were noticeably higher in the ALNP group than in the control group. However, in the CEO group, these components were reduced. In the combination (ALNP+CEO) group, the MDA content was lower but remained greater than the control. Additionally, compared to the control value, the combination group's PC level was greatly modified as represented in Table 5.

**Figure 3:**
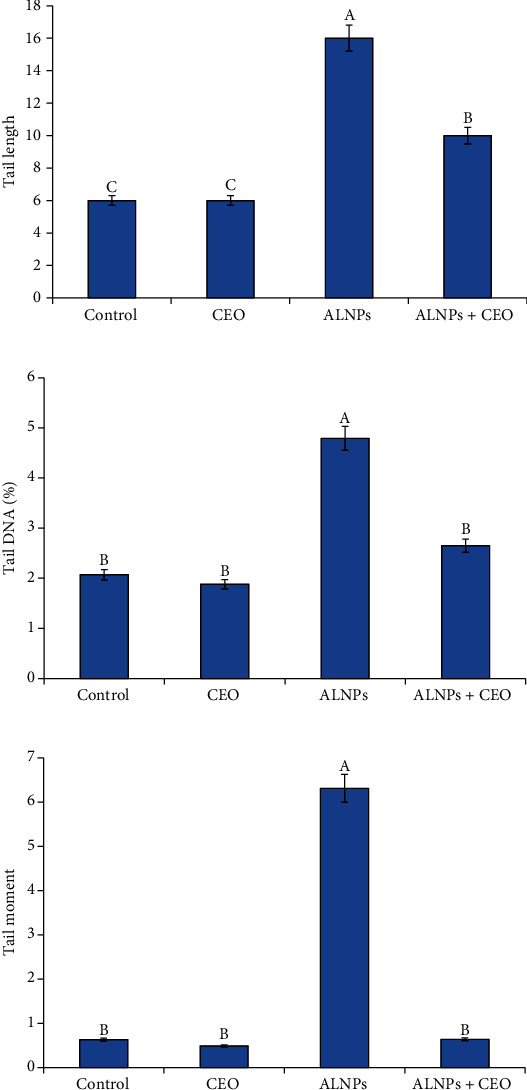
Effect of CEO (2 g/kg diet) on the comet variables in the brain of *O. niloticus* exposed to BF ALNPs (5.08 mg/L) for 30 days. Values are mean ± SE; bars are not sharing a common superscript letter (A-C) and differ significantly at *p* < 0.05.

There was a noticeable rise in the DNA damage variables in the ALNP group compared to the control. However, CEO supplementation demonstrated a considerable improvement in indicators of DNA damages compared to the ALNP group. Although the tail moment returned to the control level, the tail lengths and tail DNA% remained greater than the control as seen in Figure 3.

### 3.4. Brain Neurochemistry

#### 3.4.1. Brain Na^+^/K^+^-ATPase and AChE Enzyme Activities

As depicted in Table 6, fish exposed to ALNPs had considerably lower acetylcholinesterase and Na^+^/K^+^-ATPase levels than the control group. These activities were enhanced by the CEO cosupplementation, but not to normal levels.

#### 3.4.2. Brain and Serum Neurotransmitters

Regarding monoamine neurotransmitters, there was a substantial reduction in its levels in the ALNP group relative to the control group. In the combination group, there was a discernible improvement in serotonin levels. However, the dopamine and norepinephrine levels were unaffected by CEO supplementation. The ALNP group's GABA concentration was lower than that of the control while CEO/ALNPs combination had no appreciable impact on the GABA level (Table 6).

Concerning amino acid neurotransmitters, the ALNP group showed a substantial drop compared to the control and CEO drastically improved the concentrations of these neurotransmitters. Glycine was returned to the control amount; however, glutamine and aspartate were not (Table 6).

### 3.5. Changes in Transcriptional Profiles of Antioxidants, Stress, and Proinflammatory Genes

**Figure 4:**
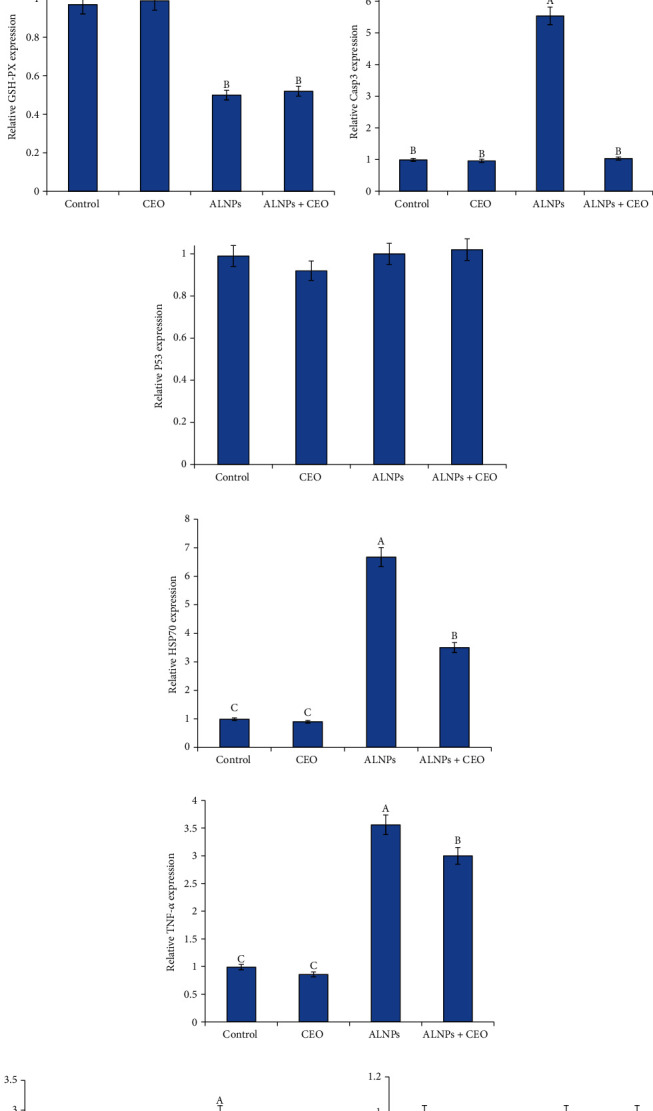
Effect of supplementation of CEO (2 g/kg diet) on the expression pattern of antioxidant ((a) SOD, (b) CAT, and (c) GSH-PX), apoptosis ((d) Casp3 and (e) P53), stress ((f) HSP70), and proinflammatory cytokine ((g)TNF-*α*, (h) IL-1*β*, and (i) INF-*γ*)-related genes in the brain of *O. niloticus* exposed to ALNPs (5.08 mg/L) for 30 days. Values are mean ± SE; bars are not sharing a common superscript letter (A-C) and differ significantly at *p* < 0.05.

Concerning antioxidant-encoding genes, SOD, CAT, and GSH-Px mRNA expressions significantly decreased in the ALNP-exposed group compared to the control. However, the coadministration of CEO with ALNPs significantly improved the expression of SOD and CAT to the control value but not for GSH-Px (Figure 4).

mRNA expressions of caspase-3 and HSP70 were significantly upregulated in fish exposed to ALNPs in comparison with the controls. CEO coadministration modulated caspase-3's pattern to the control values, but not HSP70's. Moreover, P53's mRNA expression level did not substantially differ between all the treated groups (Figure 4).

Regarding proinflammatory cytokines, TNF-*α* and IL-1*β* expression levels in the ALNP group were significantly higher than those in the control one. The expressions of these genes showed a slight improvement in the CEO/ALNP combination group but did not reach the normal. On the other hand, across all of the treated groups, the level of IFN-*γ* expression did not substantially alter (Figure 4).

### 3.6. Bioaccumulation of ALNP Residues in the Brain

**Figure 5:**
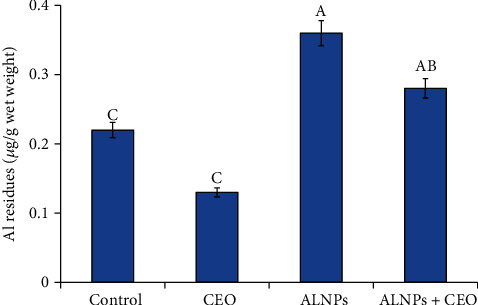
Bioaccumulation of aluminum residues in the brain tissue of the studied groups. Control group values are mean ± SE. Values among groups are not sharing a common superscript letter (a and b) and differ significantly at *p* < 0.05.

Our findings showed a significant increase in ALNP residues in brain tissues in the ALNP group. Furthermore, cosupplementation of CEO significantly decreased ALNP residues while it was still higher than the control as presented in Figure 5.

## 4. Discussion

Aquatic pollution and its effects on the ecosystem represent one of the world's current environmental problems. Nanoparticles have drawn a lot of attention in recent years since they are produced and used in a variety of different products; therefore, they quickly attained the rank as an environmental toxins. The existing study estimated the potentially toxic impacts of one of the metal oxide nanoparticles, Al_2_O_3_NPs, on the brain of freshwater fish, *Oreochromis niloticus*.

Regarding neurobehavioral changes, *O. niloticus* exposed to ALNPs showed impairments in their behavioral responses as a reduction of comfort behavior and an increase of aggressiveness. CEO coadministration with ALNPs enhanced the comfort behavior markers and decreased aggression in a dose-dependent manner, which might be owing to the ability of CEO to restore the brain's biochemical parameters. Silveira et al. [36] revealed that CEO has been reported to give a relaxing, calming, and anxiolytic effect in adult zebrafish, and these effects might be returned to the synergistic action of the ingredients in this oil. Chamomile also has a remarkable neuroprotective impact. In the rat hippocampus, its extract enhances IL-1*β* and modifies cholinergic activity while restoring scopolamine and decreasing brain-derived neurotrophic factor [37].

The antioxidant defence system enzymes can be suppressed when the system is deficient or stimulated by increased ROS generation as a defence way to counter oxidative stress [38]. In this regard, our findings declared that ALNPs induced oxidative damage in the exposed fish brain. CAT, SOD, and GSH activities were significantly lowered in the ALNP group, reflecting a deficit in protection against ROS. In the same way, De et al. [39] established that the Swiss albino mouse's tissues showed alterations in CAT and SOD activities in response to low dosages of ALNPs in its oxide form. The accumulation of ROS and free radicals resulting from changes in enzyme activity can harm biomolecules like proteins and lipids [40], leading to protein oxidation, polyunsaturated fatty acid peroxidation, and the initiation of DNA damage. Increased MDA and enhanced PC formation are consistent with decreased GSH, SOD, and CAT activities. These outcomes are consistent with the results described by different researchers for ALNPs in various biological models, where peroxidation of lipid is shown with elevated MDA level and protein carbonylation when there is a discrepancy between ROS generation and activities of antioxidant enzymes [3, 41, 42].

At this point, CEO markedly counterbalances the oxidative injury caused by ALNPs. This illustrates CEO's ability to protect against oxidative damage. It has been demonstrated that the chamomile plant's volatile oil, total flavonoids, and polysaccharides may scavenge free radicals. Additionally, mice treated with chamomile extract had higher SOD and GSH-PX activities and lower MDA levels [16]. These results provide a scientific base for the antioxidant properties of chamomile.

According to our findings, fish exposed to ALNPs had considerably lower levels of AChE and Na^+^/K^+^-ATPase. In this regard, it is recognized that ALNPs decrease the Na^+^K^+^-ATPase activity in the gills of zebrafish, leading to impaired ion regulatory activity [43]. Regarding monoamine neurotransmitters, there is a significant decrease in its levels in the ALNP group. These outcomes were in the same line with Shrivastava et al. [44] who found that accumulations of ALNPs in the brain resulted in the altered synthesis of some neurotransmitters in neurons, leading to neural impairment. Herein, CEO supplementation resulted in significant modulation in the serotonin level as chamomile and its oil has antidepressant activity and is considered excellent relievers when patients with depression have psychological and physical discomforts [45].

ROS accumulation may result in apoptosis initiation [46]. Apoptosis often causes a drop in ATP level by lowering the potential of the mitochondrial membrane. Caspase-3 mRNA expression was significantly upregulated in fish exposed to ALNPs. Moreover, ALNPs induced mitochondria-dependent apoptosis. Liu et al. [47] stated that the changes in the Bax/Bcl-2 ratios activate caspases and result in apoptosis. The elevated pattern of the caspase-3 gene was decreased when CEO was administered to ALNP-intoxicated fish. The antiapoptotic activity of CEO was revealed by Srivastava and Gupta [48] who reported that chamomile inhibits cell growth and induces apoptosis in several human cancer cell lines.

It has been observed that the levels of oxygen free radicals, particularly O^−2^, and HSP70 rise in response to stressful conditions [49]. Metal nanoparticles' toxicity is recognized to induce metabolic activities and oxidative stress in addition to peroxidation of macromolecules including proteins, lipids, and DNA. The interaction of nanoparticles is seen in the cell structure of aquatic species. Nanoparticles can readily pass the nuclear membrane so that they can engage with the vital DNA [50]. In the current study, Nile tilapia fish exposed to ALNPs elicited significant upregulations of HSP70 mRNA expression as a response to cellular stress. Our results were in the same line with those of Temiz and Kargın [5] who reported that the HSP70 levels in response to Al_2_O_3_NPs in *Oreochromis niloticus*' liver were elevated compared to the control for 3 days.

It is possible that persistent and severe oxidative stress activates inflammation-related genes and transcription factors [51]. In the existing study, ALNPs caused oxidative damage that led to subsequent inflammatory processes in the brain tissue and the expressions of TNF-*α* and IL-1*β* were markedly upregulated. Simultaneous CEO supplementation with ALNP exposure resulted in substantial improvement in these cytokine expression levels. According to Yuan et al. [52] in animal models, CEO has strong anti-inflammatory properties that suppress the release of inflammatory mediators as TNF-*α* and IL-1*β*. As shown by our GC-MS analyses, the flavonoids in chamomile are believed to be the source of the CEO's anti-inflammatory properties.

## 5. Conclusion

The current study revealed that *O. niloticus* had neurobehavioral alterations marked by extreme discomfort and aggression in response to ALNP exposure. Along with reduced Na^+^/K^+^-ATPase and AChE activities, there were lower levels of GABA, brain monoamines, and serum amino acid neurotransmitters. Antioxidant enzyme activities were also decreased in the brain whereas DNA breakage and oxidative damage were both increased with the overexpression of the brain-stress related genes HSP70, Caspase-3, pro-inflammatory cytokines in the brain tissue. Noticeably, coadministration of CEO improved the neurotoxic effect of ALNPs which was confirmed by improvements in the neurobehavioral parameters, neurochemistry analysis, antioxidant status, oxidative stress indicators, and transcripts of genes related to inflammation and stress. We collectively suggest including CEO in fish feeds to reduce the effects of any environmental toxins on the fish. Also, it is necessary to conduct further research to evaluate the underlying mechanism of each component of chamomile essential oil.

## 6. Limitations and Future Outlook

In fact, various studies have reported the possible toxic impacts of ALNPs, both *in vivo* and *in vitro*. Therefore, studying the mechanisms by which ALNPs can interact and affect the cells of biological systems becomes an issue of significance to provide a better understanding of the potential risk associated with the future uses of ALNPs in industrial and agricultural fields. Moreover, the use of some natural resources such as essential oils with powerful antioxidants and immunostimulating activities may provide promising alternatives to antibiotics which disturb the immune response of living organisms and increase the problem of antibiotic-resistant bacteria.

## Figures and Tables

**Table 1:** Retention times and peak areas (%) of the natural constituent in CEO analyzed by GC-MS.

No.	Bioactive chemical constituents	RT (min)	Peak area (%)
1	E-2-Undecen-1-ol	14.27	0.47
2	Valeric acid, 4-tridecyl ester	15.63	0.98
3	-Octanol, 2,7-dimethyl-	15.63	0.98
4	Ether, heptyl hexyl	15.63	0.98
5	*cis*-á-Farnesene	17.45	0.68
6	*trans*-á-Farnesene	17.45	0.68
7	Farnesol	17.45	0.68
8	2-Furanmethanol	22.23	0.23
9	Acetic acid	22.23	0.23
10	2H-Pyran-3-ol	24.41	3.83
11	1,6-Dioxaspiro[4.4]non-3-ene	27.14	1.48
12	Malononitrile	27.14	1.48
13	Hexadecanoic acid (CAS)	29.18	14.98
14	l-(+)-Ascorbic acid	29.18	14.98
15	2,6-Dihexadecanoate	29.18	14.98
16	6-Octadecenoic acid	32.2	62.64
17	Oleic acid	32.92	62.64

**Table 2:** Formulation and calculated composition analysis of the basal diet fed to experimental *O. niloticus* fish.

Items	Control
Ingredient (%)	
Yellow corn	210
Soybean meal 48% CP	200
Fish meal	150
Corn gluten 60% CP	130
Rice bran	110
Wheat middlings	150
Premix-min^∗^	10
Premix-vit^∗∗^	10
Corn oil	30
Total	1000
Calculated composition	
Crude protein (%)	320.5
Lipid (%)	45.50
Crude fiber (%)	42.45
Ash (%)	73.01
Nitrogen-free extract^∗∗∗^	518.54

^∗^Composition of mineral premix kg^−1^: manganese, 53 g; zinc, 40 g; iron, 20 g; copper, 2.7 g; iodine, 0.34 g; selenium, 70 mg; cobalt, 70 mg; and calcium carbonate as a carrier up to 1 kg. ^∗∗^Composition of vitamin premix kg^−1^: vitamin A, 8,000,000 IU; vitamin D3, 2,000,000 IU; vitamin E, 7,000 mg; vitamin K3, 1,500 mg; vitamin B1, 700 mg; vitamin B2, 3,500 mg; vitamin B6, 1,000 mg; vitamin B12, 7 mg; biotin, 50 mg; folic acid, 700 mg; nicotinic, 20,000 mg; and pantothenic acid, 7,000 mg. ^∗∗∗^Nitrogen-free extract = 100 − (crude protein + crude lipids + ash + crude fiber).

**Table 3:** Behavior of *O. niloticus* exposed to different ALNP concentrations for 96 h.

Behavior	Experimental groups (ALNPs (mg/L))					
	Control (0)	10	20	40	80	160
Gulping of air	—	—	++	+++	+++	++++
Distress respiration	—	—	++	+++	+++	++++
Sluggish motion	—	—	—	+++	++	+++
Abnormal swimming	—	—	+	++	+++	+++
Hyperventilation	—	—	—	+++	+++	++++

-: none; +: mild; ++: moderate; +++: strong; ++++: very strong.

**Table 4:** Effect of CEO (2 mg/kg diet) on the behavior of *O. niloticus* following ALNP (1/10th of 96 LC_50_ (5.08 mg/L)) exposure for 30 days.

Behavior	Control	CEO	ALNPs	ALNP+CEO	*p* value
Comfort behavior					
Chafing (%)	37.97 ± 0.55^a^	38.09 ± 0.06^a^	12.24 ± 1.01^c^	21.56 ± 0.29^b^	<0.001
Resting (%)	22.28 ± 0.61^b^	25.40 ± 0.34^a^	10.77 ± 0.82^d^	19.12 ± 0.36^c^	<0.001
Surfacing (%)	20.37 ± 0.19	20.34 ± 0.19	20.37 ± 0.27	20.53 ± 0.09	0.907
Schooling (%)	9.68 ± 0.09^a^	9.69 ± 0.05^a^	6.59 ± 0.06^b^	9.64 ± 0.06^a^	<0.001
Elimination (%)	10.50 ± 0.06	10.57 ± 0.09	10.43 ± 0.20	10.60 ± 0.10	0.790
Total comfort act (%)	91.94 ± 1.80^a^	92.67 ± 1.20^a^	55.80 ± 0.25^c^	60.37 ± 0.32^b^	<0.001
Aggressive behavior (%)					
Chasing (%)	5.53 ± 0.02^b^	5.17 ± 0.33^b^	7.87 ± 0.34^a^	5.49 ± 0.17^b^	<0.001
Fin tugging (%)	2.47 ± 0.22	2.45 ± 0.22	2.50 ± 0.24	2.50 ± 0.24	0.997
Mouth push (%)	8.79 ± 0.40^c^	7.79 ± 0.42^c^	13.50 ± 0.09^a^	10.42 ± 0.22^b^	<0.001
Total aggressive act (%)	17.56 ± 0.04^c^	16.36 ± 0.11^d^	22.52 ± 0.08^a^	18.18 ± 0.04^b^	<0.001

Values are mean ± SE; values in the same row are not sharing a common superscript letter (a, b, c, and d) and differ significantly at *p* < 0.05.

**Table 5:** Effect of CEO (2 mg/kg diet) on oxidative status of *O. niloticus* following ALNP (1/10th of 96 LC_50_ (5.08 mg/L)) exposure for 30 days.

	Control	CEO	ALNPs	ALNP+CEO	*p* value
Antioxidant markers					
SOD (U/g tissue)	7.66 ± 0.12^b^	8.92 ± 0.03^a^	3.35 ± 0.13^d^	5.56 ± 0.28^c^	<0.001
CAT (U/g tissue)	5.43 ± 0.11^b^	7.26 ± 0.12^a^	4.07 ± 0.06^c^	5.38 ± 0.03^b^	<0.001
GSH (nmol/g tissue)	11.15 ± 0.03^b^	12.14 ± 0.04^a^	7.72 ± 0.43^c^	11.15 ± 0.03^b^	<0.001
Oxidative stress biomarkers					<0.001
MDA (nmol/g tissue)	16.61 ± 0.19^c^	14.37 ± 0.33^c^	41.58 ± 2.14^a^	27.89 ± 0.15^b^	<0.001
PC (nmol/g tissue)	6.02 ± 0.02^b^	5.50 ± 0.18^b^	13.27 ± 0.29^a^	6.03 ± 0.01^b^	<0.001


Values are mean ± SE; values in the same row are not sharing a common superscript letter (a, b, c, and d) and differ significantly at *p* < 0.05.

**Table 6:** Effect of CEO (2 mg/kg diet) on brain neurochemistry of *O. niloticus* following ALNP (1/10th of 96 LC_50_ (5.08 mg/L)) exposure for 30 days.

	Control	CEO	ALNPs	ALNP+CEO	*p* value
AChE and monoamine neurotransmitters					
AChE (ng/mg protein)	75.20 ± 0.06^a^	75.22 ± 0.59^a^	26.09 ± 0.72^c^	55.34 ± 2.72^b^	<0.001
NA-ATPase (ng/mg protein)	19.51 ± 0.29^a^	19.51 ± 0.29^a^	15.11 ± 0.07^b^	18.51 ± 0.09^a^	<0.001
Serotonin (ng/g tissue)	6.55 ± 0.43^a^	6.54 ± 0.43^a^	3.59 ± 1.23^b^	7.47 ± 0.14^a^	0.019
Dopamine (ng/g tissue)	3.07 ± 0.03^a^	3.25 ± 0.14^a^	1.12 ± 0.19^c^	2.11 ± 0.07^b^	<0.001
Norepinephrine (ng/g tissue)	0.65 ± 0.00^a^	0.65 ± 0.01^a^	0.62 ± 0.00^b^	0.63 ± 0.01^ab^	0.011
GABA (ng/g tissue)	500.15 ± 0.04^a^	500.08 ± 0.04^a^	450.37 ± 8.40^b^	455.00 ± 2.89^b^	<0.001
Amino acid neurotransmitters					<0.001
Glycine (nmol/mL)	3.67 ± 0.291^a^	3.83 ± 0.30^a^	2.47 ± 0.54^b^	3.33 ± 0.30^a^	<0.001
Aspartic acid (nmol/mL)	1.21 ± 0.69^a^	1.45 ± 0.70^a^	0.90 ± 0.10^c^	1.07 ± 0.30^b^	<0.001
Glutamic acid (nmol/mL)	3.43 ± 0.30^b^	3.74 ± 0.27^a^	0.92 ± 0.11^d^	2.06 ± 1.05^c^	<0.001

Values are mean ± SE; values in the same row are not sharing a common superscript letter (a, b, c, and d) and differ significantly at *p* < 0.05.

## Data Availability

The data presented in this study are available on request from the corresponding author.
